# Concomitant food intake markedly alters plasma glutamic acid kinetics after oral monosodium glutamate administration in rats: Relevance to dietary safety evaluation

**DOI:** 10.1016/j.toxrep.2025.102191

**Published:** 2025-12-16

**Authors:** Ryosei Sakai, Risa Motoi, Yusuke Amino, Kohsuke Hayamizu

**Affiliations:** Laboratory of Food Chemistry, Yokohama University of Pharmacy, Yokohama, Japan

**Keywords:** Monosodium glutamate (MSG), Glutamic acid, Plasma kinetics, Food co-ingestion, Dietary exposure, Toxicological relevance, Food additive, Rats

## Abstract

**Background:**

Monosodium glutamate (MSG) is widely used as a flavor enhancer and has been evaluated as safe by international authorities. However, some toxicological studies have employed oral bolus dosing of MSG without food, an approach that is unlikely to reflect physiological dietary exposure.

**Objectives:**

To determine how concomitant food intake modifies plasma glutamic acid kinetics following oral MSG administration in rats, thereby improving the interpretation of toxicological data.

**Methods:**

Male Wistar rats received graded oral doses of MSG (150, 300, or 600 mg/kg) with or without a liquid diet (Sustagen®). Plasma glutamic acid concentrations were measured by LC-MS/MS, and pharmacokinetic parameters were determined.

**Results:**

Bolus MSG administration alone caused rapid, dose-dependent increases in plasma glutamic acid, with peak concentrations occurring 20–30 min post-dose. Co-administration of Sustagen® markedly reduced both C_max_ and AUC and also delayed T_max_, indicating that food intake substantially attenuated systemic glutamic acid exposure.

**Conclusions:**

Concomitant food intake profoundly alters plasma glutamic acid kinetics after oral MSG administration in rats. These findings emphasize the importance of considering realistic dietary exposure conditions when interpreting toxicological studies of MSG and related glutamate salts and underscore the need for physiologically relevant dosing regimens.

## Highlights

• Food co-ingestion markedly suppressed the plasma glutamic acid rise after oral MSG, reducing C_max_ and AUC across doses.
• Bolus MSG alone produced rapid, dose-dependent plasma glutamic acid elevations.
• Co-ingestion lowered systemic exposure and delayed T_max_, altering the plasma appearance profile.
• Findings underscore the importance of physiologically relevant dosing conditions in MSG safety evaluation.

## Graphical Abstract

**Figure:**
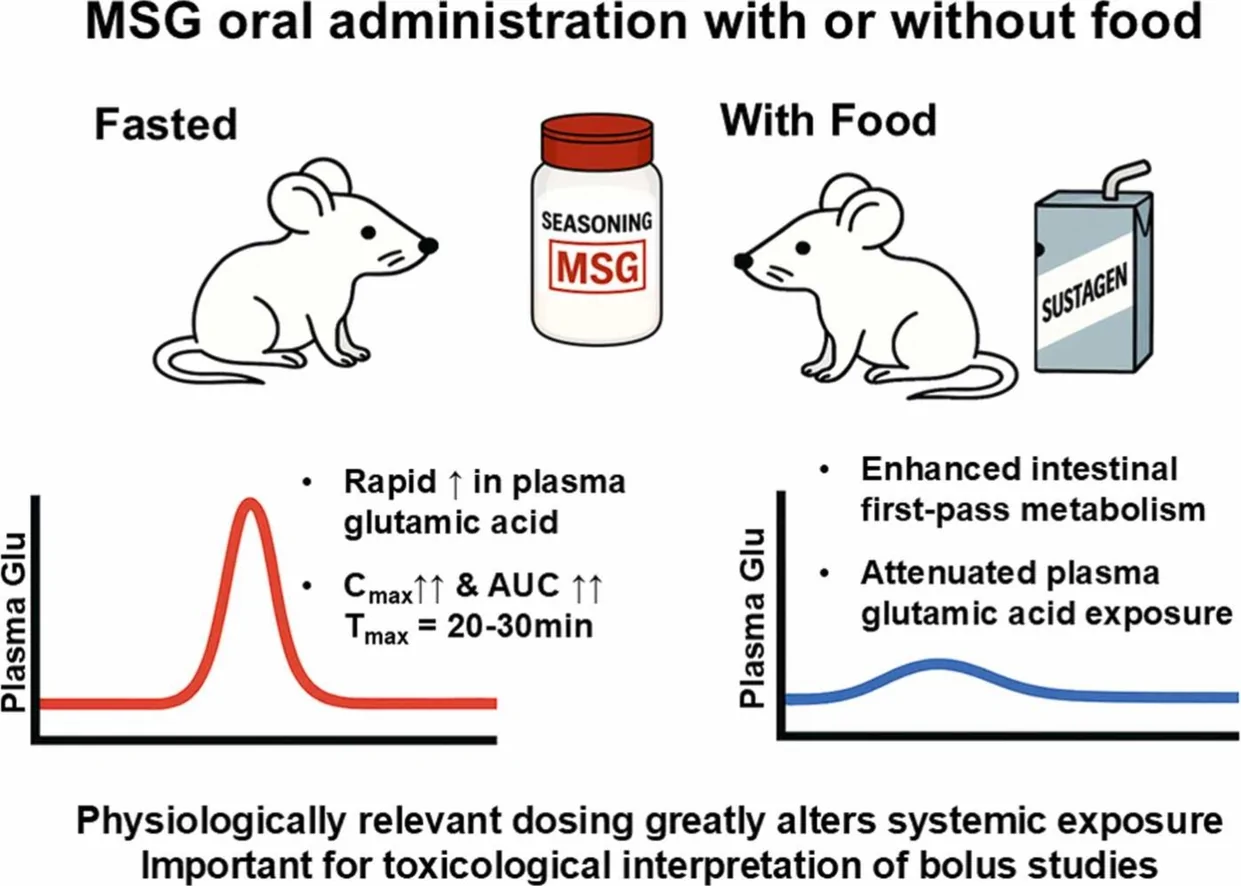

## 1 Introduction

Glutamic acid is one of the most abundant amino acids in foods. Amino acid analyses of food protein hydrolysates show that glutamic acid accounts for approximately 10–20 % of animal proteins and 10–40 % of plant proteins. In the United States, the average daily intake of glutamic acid (including glutamine) in adult males is about 20 g/day, corresponding to roughly 20 % of total amino acid intake [1]. In Germany, dietary intake of glutamic acid (excluding glutamine) has been estimated to reach about 10 g/day [2].

In addition to its protein-bound form, free glutamic acid is abundant in various foods such as meat, tomatoes, human breast milk, and traditional fermented foods including miso, soy sauce, and cheese. It enhances palatability through its characteristic umami taste [3]. The average daily intake of free glutamic acid from natural foods is approximately 1 g/day [2]. Furthermore, crystalline glutamic acid and its salts (e.g., monosodium glutamate (MSG)) are widely used as seasonings and flavor enhancers.

Ingested glutamic acid is primarily utilized for energy production and for the synthesis of other amino acids such as alanine, proline, and citrulline during first-pass metabolism in the intestinal tract [4], [5], [6], [7], and glutamic acid derived from MSG follows the same metabolic pathways, as noted by European Food Safety Authority (EFSA) [8]. Because plasma glutamic acid concentrations (i.e. extracellular levels) are tightly regulated, dietary glutamic acid, even when consumed in large amounts, does not disturb neurotransmitter signaling. The long history of dietary consumption supports its safety, and the use of added glutamic acid and its salts as food additives has also been considered safe for more than a century.

International authorities, including the Joint FAO/WHO Expert Committee on Food Additives (JECFA)[9], the U.S. Food and Drug Administration (FDA) [10], and EFSA [8], have evaluated MSG and related glutamate salts based on extensive toxicological and dietary exposure data. The FDA recognizes MSG as “generally recognized as safe” (GRAS), and JECFA concluded that an Acceptable Daily Intake (ADI) was “not specified,” indicating no safety concern at levels used in food. Earlier European evaluations, including those by the Scientific Committee for Food [11], reached a similar conclusion, although EFSA’s more recent scientific opinion [8] provisionally established a group ADI of 30 mg/kg body weight per day (expressed as glutamic acid) based on available toxicological data, while emphasizing the importance of considering differences between bolus and dietary exposure conditions when interpreting study results [12]. These international evaluations collectively underscore the importance of understanding physiological exposure contexts when interpreting toxicological data.

Previous human studies have shown that plasma glutamic acid levels rise after bolus ingestion of MSG alone but not when MSG is consumed with food [13], and similar attenuation with food has been reported in mice and monkeys [14]. Rats remain the primary species used in regulatory toxicological studies of MSG and related glutamate salts; however, the influence of concomitant food intake on plasma glutamic acid kinetics has not been characterized in this species. Therefore, the present study aimed to examine plasma glutamic acid kinetics in rats following oral MSG administration with or without concomitant food intake, and thereby clarify how bolus dosing versus dietary co-ingestion influences the interpretation of toxicological findings specifically for MSG.

## 2 Materials and methods

### 2.1 Animals and experimental design

The study protocol was approved by the Institutional Animal Care and Use Committee of Mediford Corporation (Japan). Twelve-week-old male Wistar rats purchased from The Jackson Laboratory Japan were maintained under controlled environmental conditions (12-h light/dark cycle, 19–25 °C, and 35–75 % humidity) and fed a standard pellet diet with free access to water. After overnight fasting, rats were divided into six groups (n = 6 per group) based on body weight. Graded doses of MSG (150, 300, or 600 mg/kg; equivalent to 0.80, 1.60, or 3.20 mmol/kg, respectively; MSG: L-glutamic acid monosodium salt monohydrate, > 99.0 %, Sigma-Aldrich) were orally administered by gavage at a dose volume of 20 mL/kg body weight, with or without a liquid diet (Sustagen®, Nestlé Health Science; 94 kcal/dL, 5.8 g protein/dL). For the co-ingestion condition, MSG was completely dissolved in Sustagen® immediately before dosing, and the mixture was given by gavage. Blood samples were collected from the subclavian vein at 0, 10, 20, 30, 45, 60, and 90 min after dosing. Plasma was separated using sodium heparin as an anticoagulant and stored at −80 °C until analysis.

### 2.2 Measurement of glutamic acid concentration

Plasma glutamic acid concentrations were determined by liquid chromatography–tandem mass spectrometry (LC-MS/MS; Agilent 1200 Series LC coupled with a 6400 Series Triple Quadrupole) following the sample preparation, derivatization, and analytical procedures described by Nakamura et al. [15]. One volume of plasma was deproteinized with four volumes of methanol containing 20 µM [U-^13^C]glutamic acid as an internal standard. The supernatant was derivatized with 6-aminoquinolyl-N-hydroxysuccinimidyl carbamate (AQC) and analyzed using an L-column ODS column. Positive ions generated by electrospray ionization (ESI) were analyzed in multiple reaction monitoring (MRM) mode. The precursor ions (*m/z* = 318 for natural glutamic acid and 323 for the internal standard) and product ion (*m/z* = 171) were monitored. The ratio of natural to [U-^13^C]glutamic acid peak areas was used to calculate plasma concentrations.

### 2.3 Calculations

Pharmacokinetic parameters (C_max_, T_max_, incremental C_max_ (iC_max_), AUC, incremental AUC (iAUC), T_1/2_, and Ke) were calculated using the MOMENT (Excel-based) program [16]. The apparent absorption rate (µM/min) was estimated as the slope of the concentration–time curve between 0 and T_max_ by least-squares regression. Incremental parameters (iC_max_ and iAUC) were used to account for basal plasma glutamic acid levels and to isolate the response attributable to MSG administration.

### 2.4 Statistical analysis

All data are expressed as mean ± standard deviation (SD). Between-group comparisons were performed using independent-sample *t*-tests, and within-group analyses were performed using paired *t*-tests with Bonferroni correction. Plasma concentration-time data were analyzed using repeated-measures ANOVA. Statistical significance was defined as p < 0.05. All statistical analyses were performed using R (v4.4.4).

## 3 Results

Plasma glutamic acid concentrations increased in a dose-dependent manner after oral MSG administration without food, reaching peak values at 20–30 min post-dose (Fig. 1). Co-administration of Sustagen® markedly attenuated these elevations across all dose levels.

**Fig. 1:**
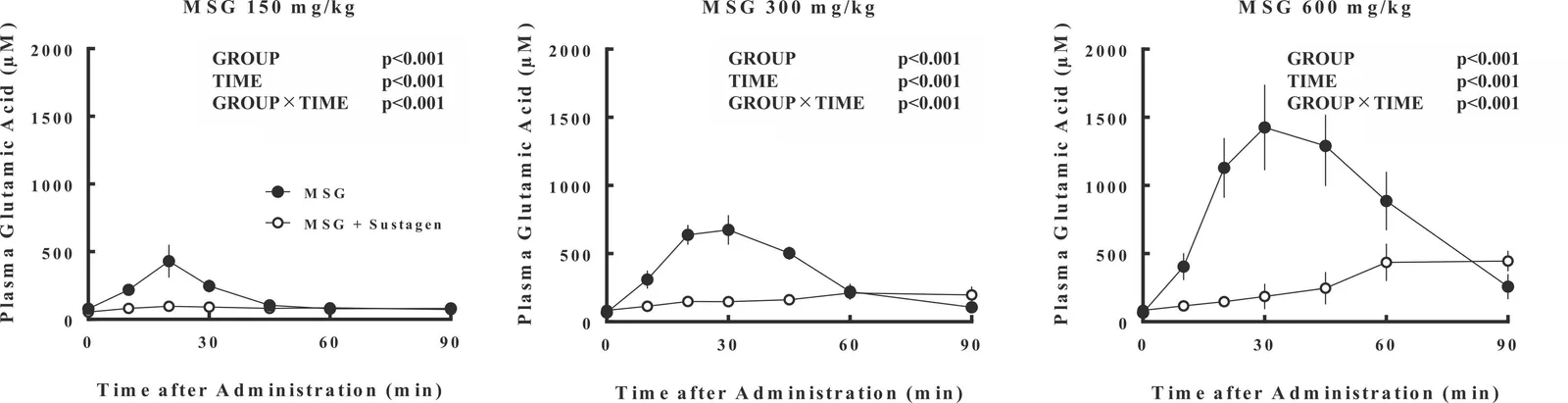
Plasma glutamic acid concentrations after oral administration of MSG with or without Sustagen. Graded doses of MSG (150, 300, or 600 mg/kg) were orally administered with (open circles) or without (closed circles) Sustagen®. Mean plasma glutamic acid concentrations at each time point after MSG administration are plotted. Error bars represent standard deviations (SDs). Statistical analysis was performed using repeated-measures ANOVA. GROUP: effect of Sustagen® co-administration; TIME: effect of sampling time; GROUP × TIME: interaction between Sustagen and time (all p < 0.001).

C_max_ and iC_max_ increased linearly with MSG dose under fasting conditions but were markedly reduced by co-administration of Sustagen®. The reductions in iC_max_ were 87 %, 78 %, and 69 % for 150, 300, and 600 mg/kg MSG, respectively (Table 1 and Fig. 2). Comparable dose-dependent reductions were observed for both AUC and iAUC values; Sustagen® decreased iAUC by 67–75 % across doses.

**Table 1:** Pharmacokinetic parameters of plasma glutamic acid after oral administration of monosodium glutamate (MSG) with or without Sustagen® in rats.

	Plasma concentrations (μM)								
	150 mg MSG / kg			300 mg MSG / kg			600 mg MSG / kg		
	Sustagen (-)(n = 6)	Sustagen (+)(n = 6)	p-value	Sustagen (-)(n = 6)	Sustagen (+)(n = 6)	p-value	Sustagen (-)(n = 6)	Sustagen (+)(n = 6)	p-value
T_max_ (min)	20 ± 0	18 ± 8	0.611	25 ± 5	70 ± 15	< 0.001	35 ± 8	70 ± 15	< 0.001
C_max_ (μM)	430 ± 121	102 ± 18	0.001	712 ± 87	224 ± 55	< 0.001	1450 ± 312	507 ± 37	< 0.001
AUC (µM･min)	14496 ± 1462	7419 ± 1083	< 0.001	32382 ± 2776	15031 ± 2578	< 0.001	76619 ± 14920	25568 ± 4817	< 0.001
iC_max_ (µM)	351 ± 120	47 ± 19	0.001	643 ± 91	139 ± 51	< 0.001	1383 ± 313	423 ± 33	< 0.001
iAUC (µM･min)	7381 ± 2371	2483 ± 1192	0.001	26166 ± 2875	7441 ± 2563	< 0.001	70579 ± 14349	17956 ± 4483	< 0.001
T_1/2_ (min)	17 ± 3	75 ± 23	0.001	23 ± 3	ND		22 ± 7	ND	
Ke (/min)	0.044 ± 0.009	0.010 ± 0.002	< 0.001	0.031 ± 0.005	ND		0.034 ± 0.009	ND	

Data are presented as mean ± SD (n = 6). Maximum concentration (C_max_), time to Cmax (T_max_), incremental maximum concentration (iC_max_), area under the concentration–time curve (AUC), incremental AUC (iAUC), half-life (T_1/2_), and elimination rate constant (Ke) were calculated from the time-course data of plasma glutamic acid concentrations in individual rats. iC_max_ and iAUC were calculated as increases from baseline values. Statistical differences between groups were evaluated using the independent *t*-test. N.D., not determined due to insufficient data points for reliable estimation of the elimination phase.

**Fig. 2:**
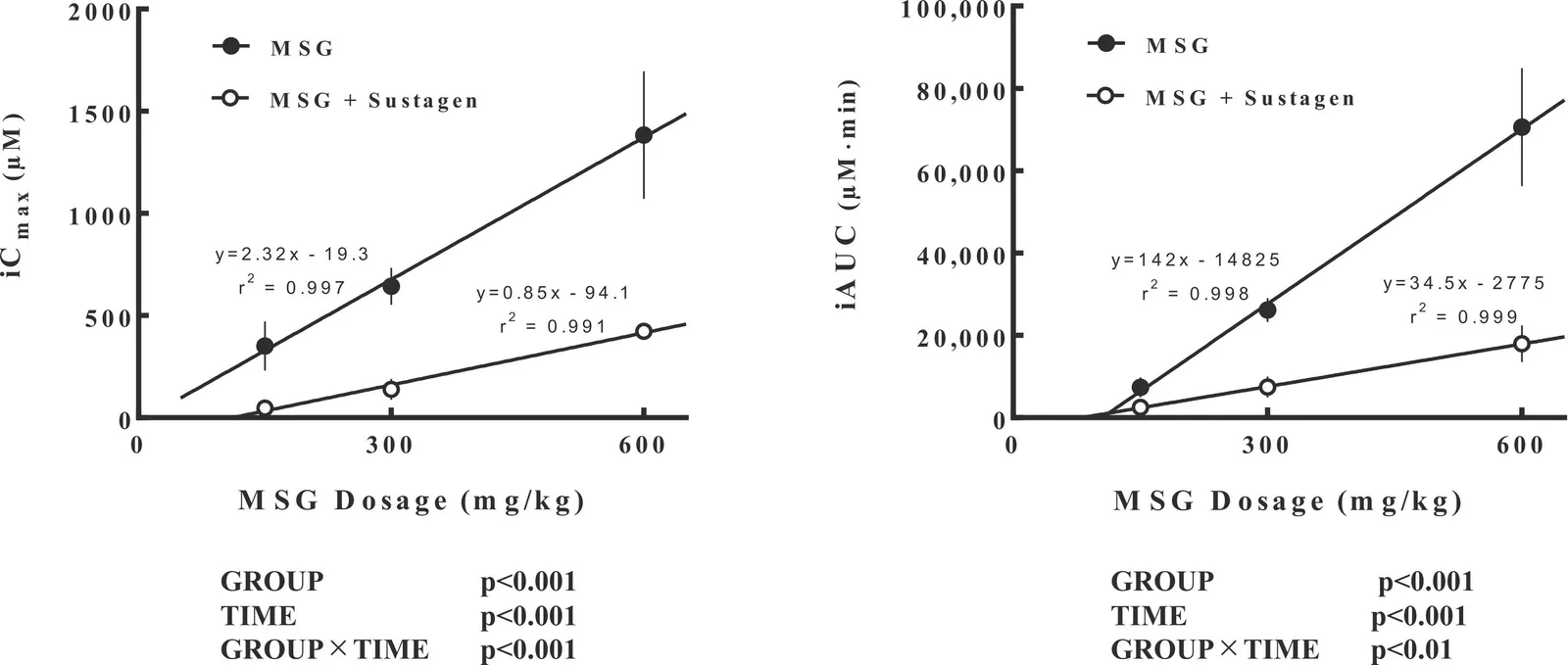
Linear relationships of iC_max_ and iAUC with MSG dose, and effects of Sustagen co-administration. Average iC_max_ (left panel) and iAUC (right panel) values for each MSG dose are plotted. Positive linear relationships between MSG dose and iC_max_ were observed in both groups receiving MSG with (open circles) and without (closed circles) Sustagen® (r² = 0.991 and 0.997, respectively). Similar correlations were observed for iAUC (r² = 0.999 and 0.998, respectively). Co-administration of Sustagen® significantly reduced both iC_max_ and iAUC. Statistical analysis was conducted using repeated-measures ANOVA. GROUP: effect of Sustagen® co-administration; TIME: effect of sampling time; GROUP × TIME: interaction between Sustagen and time. Error bars represent SDs.

The apparent net absorption rate of glutamic acid increased proportionally with dose under fasting conditions but was significantly lower with Sustagen® at all doses (Fig. 3). T_max_ was significantly delayed by co-administration of Sustagen® at higher MSG doses (Table 1).

**Fig. 3:**
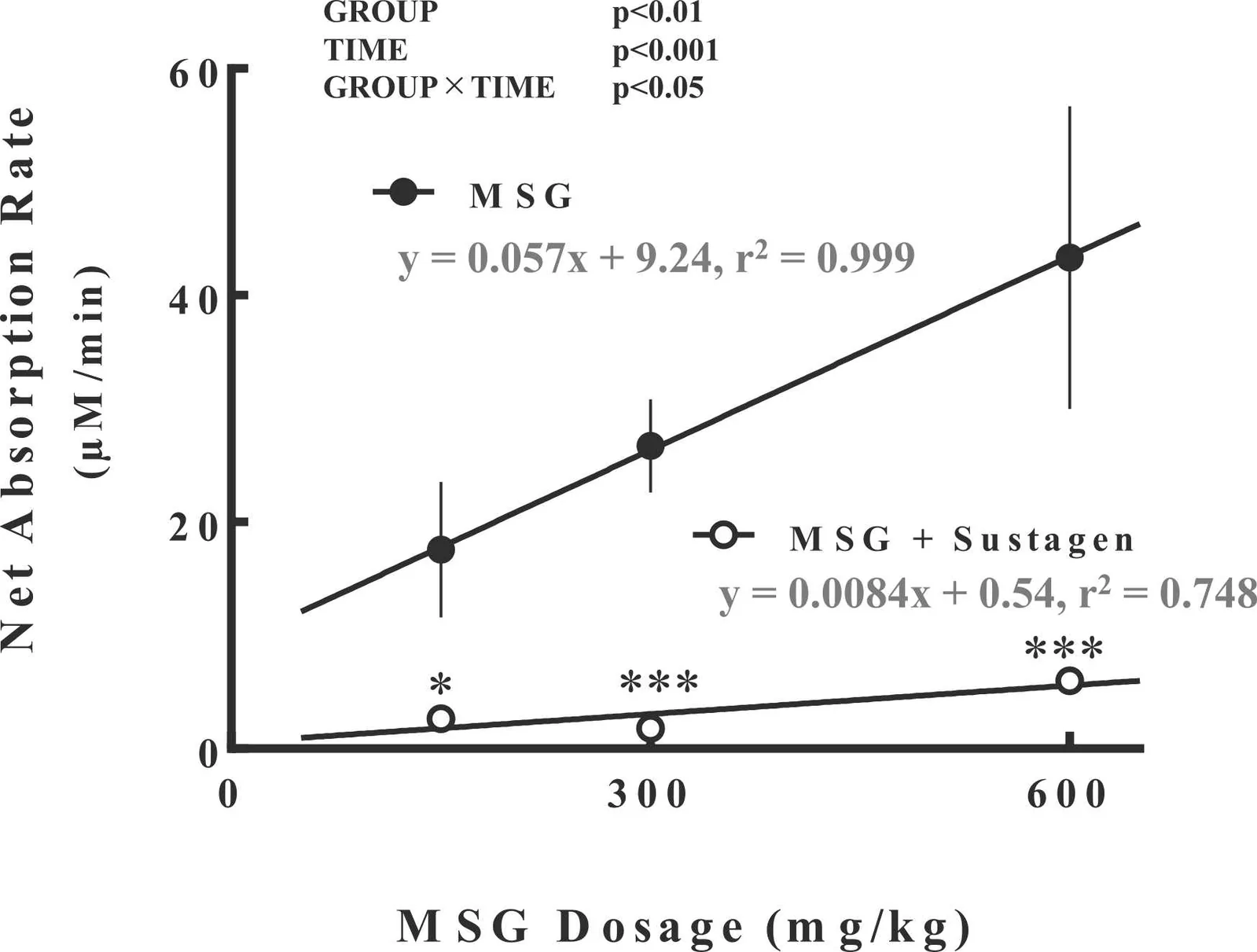
Dose-dependent changes in apparent absorption rate of plasma glutamic acid after MSG administration. Average apparent absorption rates at each MSG dose are plotted. Positive linear relationships between MSG dose and apparent absorption rate were observed in both groups receiving MSG with (open circles, r² = 0.748) and without (closed circles, r² = 0.999) Sustagen®. Co-administration of Sustagen® significantly reduced apparent absorption rates at all MSG doses. (p < 0.05, **p < 0.001 vs. without Sustagen; Student’s *t*-test with Bonferroni correction.) Repeated-measures ANOVA results: GROUP (p < 0.01), TIME (p < 0.001), GROUP × TIME (p < 0.05). Error bars represent SDs.

These findings demonstrate that co-administration of food substantially reduces the magnitude of plasma glutamic acid excursions after oral MSG administration and alters the time course of the plasma concentration profile.

## 4 Discussion

The present study demonstrated that concomitant meal ingestion markedly alters plasma glutamic acid kinetics following oral MSG administration in rats. Co-administration of a liquid diet significantly reduced both C_max_ and AUC values of plasma glutamic acid compared with MSG administered alone, indicating that food intake substantially modifies the magnitude and time course of plasma glutamic acid excursions. These findings agree with previous human, mouse, and monkey studies showing that plasma glutamic acid concentrations increase markedly after oral administration of MSG without food but are substantially attenuated when MSG is administered together with a liquid formula diet [14], [13]. The present study extends these observations to rats by examining an analogous co-administration condition in which MSG and a liquid diet were given simultaneously. In rats, comparable attenuation has been reported under a different feeding condition: when solid food was available ad libitum before bolus MSG administration, the rise in plasma glutamic acid was similarly suppressed [17].

This attenuation of plasma glutamic acid elevation under fed conditions can be explained by several physiological mechanisms. First, most dietary glutamic acid is metabolized within the intestinal mucosa before reaching the systemic circulation. Studies in rats, piglets, and preterm infants have consistently shown extensive intestinal metabolism of glutamic acid, whereby a large proportion of glutamate carbon is oxidized to CO₂ and its nitrogen is converted to other amino acids such as alanine and branched-chain amino acids [4], [5], [6], [7]. Although intestinal metabolism was not directly assessed in the present study, this established evidence offers a plausible explanation for the attenuated plasma responses observed with food co-ingestion due to the availability of other nutrients for metabolic cofactors. Indeed, the beneficial effect of carbohydrate co-ingestion in reducing plasma glutamic acid elevation has been shown in both humans [18] and rats [17]. This attenuation can be partly explained by the well-established metabolic fate of dietary glutamic acid, whose nitrogen is mainly converted to alanine through transamination with pyruvate derived from dietary carbohydrates, as summarized by Cynober [19].

Second, concomitant meal ingestion delays gastric emptying, thereby slowing the rate at which glutamic acid enters the intestine and reducing peak plasma concentrations. This delayed gastric emptying is consistent with prior reports in rats showing that liquid diets slow gastric transit relative to water [20], leading to lower and more prolonged postprandial nutrient absorption. This interpretation is compatible with the results that meal co-ingestion delayed T_max_ at higher MSG doses in the present study.

The linear increases in plasma glutamic acid parameters (iC_max_ and iAUC) with increasing dose under fasting conditions (Fig. 2) likely reflect a transient overflow beyond intestinal metabolic capacity. Since nearly all filtered amino acids, including glutamic acid, are reabsorbed in the proximal tubule [21], the slower elimination observed at higher doses is more likely attributable to capacity-limited intestinal handling rather than altered renal clearance. The increases of iC_max_ and iAUC with increasing dose were markedly reduced by concomitant meal ingestion (Fig. 2). Burrin DG et al. [22] reported that intestinal metabolic capacity for glutamic acid is substantially high when it is consumed with food, which is consistent with the attenuated dose-dependency observed here, although the specific mechanisms were not directly investigated.

The substantial differences in systemic exposure observed between bolus and co-ingestion conditions are highly relevant for the interpretation of toxicological data. Toxicity studies that employ oral bolus (gavage) administration without food may not accurately reflect physiological dietary exposure to MSG, which is invariably consumed as part of food. Plasma glutamic acid profiles under such fasting, bolus-type conditions differ substantially from those expected under normal dietary ingestion, resulting in exaggerated systemic exposures and potentially misleading NOAEL (No Observed Adverse Effect Level) determination. Therefore, caution is warranted when extrapolating such animal data to human dietary exposure.

It is noteworthy that international authorities such as JECFA, FDA, and EFSA have acknowledged the importance of considering realistic exposure conditions when evaluating the safety of food additives such as glutamate salts [24], [23], [12]. The present findings reinforce this principle, demonstrating experimentally that food co-ingestion markedly alters plasma kinetics. From a toxicological risk assessment perspective, these data support the relevance of dietary-type administration in future safety evaluations of MSG and related compounds.

In conclusion, this study provides direct evidence that concomitant food intake modifies the plasma kinetics of glutamic acid, thereby limiting systemic exposure to glutamic acid following oral MSG administration. These results emphasize the importance of considering physiologically relevant exposure conditions in toxicological assessment of MSG and related glutamate salts.

## CRediT authorship contribution statement

**Kohsuke Hayamizu:** Writing – review & editing, Supervision, Project administration. **Yusuke Amino:** Investigation. **Risa Motoi:** Investigation. **Ryosei Sakai:** Writing – review & editing, Writing – original draft, Supervision, Project administration, Conceptualization.

## Declaration of Competing Interest

The authors declare the following financial interests/personal relationships which may be considered as potential competing interests: The authors declare that the study was funded by a research grant from the International Glutamate Technical Committee (USA). The funder had no role in the study design, data collection and analysis, decision to publish, or preparation of the manuscript. All authors declare no other known competing financial interests or personal relationships that could have appeared to influence the work reported in this paper.

## Data Availability

Data will be made available on request.
